# A cross-sectional international study shows confidence in public health scientists predicts use of COVID-19 non-pharmaceutical interventions

**DOI:** 10.1186/s12889-022-13074-3

**Published:** 2022-04-06

**Authors:** Shaun Goldfinch, Ross Taplin

**Affiliations:** 1 Australia New Zealand School of Government, Carlton VIC 3053, Melbourne, Australia; 2 Curtin University, Perth, WA 6845 Australia

**Keywords:** COVID-19, Trust in government, Confidence, Public Health Scientists, Face Masks, Social distancing, Phone tracking application, Health policy

## Abstract

**Background:**

We examine the antecedents of COVID-19 phone tracking applications use, social distancing, and mask use, in the United States, Hong Kong and Japan.

**Methods:**

We draw on online panel surveys of over 1000 respondents each in the USA, Hong Kong and Japan, using broadly representative quota sample selections. Results are tested by ordinal logistic regression for the two ordinal dependent variables and logistic regression for phone app use.

**Results:**

Confidence in public health scientists predicts use of phone tracking applications, social distancing, and mask use, albeit statistically insignificant for tracer phone application use in Hong Kong. Trust in government predicts the use of a phone tracking application. Counterintuitively, trust in government is *negatively* and significantly associated with mask use and social distancing in Hong Kong and Japan. Women are more likely to wear masks and practice social distancing. Government employees are more likely to use a phone tracking application, but less likely to mask and social distance. Voting and civic participation are positively associated with trust in government and confidence in public health scientists, in all three countries. There are interesting variations across all three countries on other antecedents and controls.

**Conclusions:**

Building and maintaining confidence in public health scientists provides a key tool to manage pandemics. Credible, effectively communicative – and independent – medical and scientific leaders may be central to pandemic control success. For digital measures, trust in government and privacy protection is central. Political and social factors are important to understand successful public health policy implementation.

## Background

The COVID-19 pandemic continues without an obvious end in sight. With new COVID varieties arising, vaccine efficacy declining over time, vaccination applied unevenly around the world, and with hesitancy seeing vaccine adoption stalling even in countries with sufficient supply, non-pharmaceutical interventions (NPIs) to control the pandemic will continue to be of value [1–3]. We are interested in what factors make individuals more likely to adopt non-pharmaceutical pandemic control mechanisms, including mask use. A broader and multi-disciplinary focus on public health, social science, and policy research and practice, is perhaps useful for this task, drawing on insights in implementation and policy development for public health [4]. We draw on the public health, as well as the broader policy, literature, on social engagement, trust in government, and confidence, and their possible role in adoption of COVID-19 control mechanisms [5–9].

Goldfinch et al. found in Australia and New Zealand that trust in government and confidence in public health scientists were key predictors of the use of a COVID-19 phone tracking application, more so than socio-demographic and ideological/partisan factors [8]. Studies in the United States found that trust in government and government sources was associated with adherence to social distancing [6]. Kazemian et al. found in the United States that scientific trust raised support (although adoption was not measured) for Covid-19 social distancing policies [7]. Expanding this analysis beyond the United States of America (USA), to include Japan, and Hong Kong (HK), and focusing on the self-reported adoption of pandemic control measures including social distancing and mask wearing, and using expanded antecedents, we generate some novel, highly topical, and policy-relevant findings. A key finding is that confidence in public health scientists predicts the use of COVID-19 non-pharmaceutical interventions across all three countries. While trust in government predicts the use of a phone tracking application, it is insignificant for masking and social distancing in the USA, and negative and significant in Japan and Hong Kong. We outline other findings below.

### Hypotheses

Trust and confidence in government and official actors can predict success and/or adoption of health and other policies, policy instruments, directives, and moral suasion, including policy compliance during COVID-19 [5–9]. Studies in the United States found that trust in government and government sources was associated with adherence to social distancing [6]. COVID-19 has seen public health scientists take leading roles in pandemic control and communication, with figures such as Dr Anthony Fauci in the USA becoming national and international celebrities, suggesting scientific leadership is also important. Indeed, Kazemian et al. found in the United States that scientific trust raised support (although adoption was not measured) for Covid-19 social distancing policies [7]. Goldfinch et al. found both confidence in public health scientists and trust in government were antecedents of use of a COVID-19 phone tracking application in Australia and New Zealand [8].

Digital contact tracing to control COVID-19 raises privacy concerns [10]. However, Horvath et al. found trust in the UK National Health Service mitigates these, with the benefits of app use perceived to be larger than the risks of privacy and security breaches [11]. Oldeweme et al. found transparency, social influence and trust in government fosters tracking application adoption [12]. Expanding on these, in each country we test the following hypotheses:

- *H1:* Use of a COVID-19 phone tracer application (*H1a*), social distancing (*H1b*) and a mask (*H1c*) is higher when trust in government is higher.
- *H2:* Use of a COVID-19 phone tracer application (*H2a*), social distancing (*H2b*) and a mask (*H2c*) is higher when confidence in public health scientists is higher.

## Methods

### Survey

A commercial company, Dynata, delivered online surveys in the USA, HK, and Japan amidst the COVID-19 pandemic. A survey invitation was sent to its panel in each country to generate representative quotas based on age, gender, and region [13]. Quota sampling is widely used, including in medical, public health, and epidemiological research, and is considered state-of-the-art [14]. Surveys were translated into Mandarin and Cantonese for HK, and Japanese for Japan, with modifications for context. Panellists were incentivized through allocated points for survey completion, which accumulate towards a voucher usable for purchases. All methods were carried out in accordance with relevant guidelines and/or the Declaration of Helsinki. Over 1000 respondents (1044 USA; 1019 Japan; 1004 HK) from each country were included in the analysis. The USA sample is generally representative, with Japan and HK underrepresented for over-55s, who are less likely to be members of online panels (Table 2).

### Variables

Our independent variables are as follows. Trust in government was measured by responses to ‘Government is generally trustworthy’, on a 4-point scale from ‘strongly agree’ to ‘strongly disagree’, replicated from Goldfinch et al. [8]. Confidence in scientific expertise was measured by asking ‘How much confidence do you have that public health scientists act in the best interests of the public?’, ranging on a Likert 4-point scale from ‘A great deal’ to ‘not at all’, adapted from Pew Research Centre [15] (Table 1).

Our three dependent variables are as follows. First, whether a COVID-19 phone App was used (*App_use* measured yes/no); second, the extent social distancing was practiced (*social_dist*); and third, whether a mask (*mask*) was used in public (both on 4-point Likert scales, from ‘never’ to ‘always’) (Table 3).

Our control variables and their relationships with the independent variables are as follows. Trust/confidence, political attitudes, and behavior, are found to be conditioned by socio-demographic factors – albeit in contested directions [5–9]. For example, protective behavior adoption was found to be correlated with age and co-morbidity risk in China [16]. Females are found to be more likely to comply with government directives [17]. Older people might exhibit lower digital competence, but be more compliant with government directives [18]. We measure age, education, income, gender, and residence (urban/rural). Personal income responses were specified as ranges in local currencies for comparability with local measures (Table 2). Household income was also measured but regression results are similar, so is not reported here. Government employment (current or past) was measured as it has been found to be associated with policy compliance and pro-social behaviour [19]. Trust in security of information supplied by digital means to government is measured (*trust_info*), on the prediction that this influences the willingness to supply personal information needed for phone tracking applications as privacy is better protected (this is used only in the regressions that test this) [11, 12, 20]. Civic participation (*partic_civic*), an element of social capital, was measured as it may be associated with pro-social behaviour and positive health policy outcomes [5, 21].

Partisan and ideological factors influence policy responses and behaviour, trust in government, and attitudes and responses to COVID-19, including adoption of COVD-19 control mechanisms [8, 22, 23]. Conservative and Republican political orientation was found to be associated with less trust in science in the United States during the pandemic, while Republicans were less likely to comply with COVID-19 control measures [22–24]. We measure self-identified political ideology (*conservative*) on a scale from 1 (more left or liberal) to 5 (more right or conservative), adapted from the World Values Survey [25]. Voting/not voting is measured. Political party voting patterns are also measured, but are reported only for the United States Congressional elections as parties differ by country, and HK is a not democracy. Other voting results are available from the authors.

### Statistical Analysis

Our statistical analysis is as follows. Ordinal logistic regression was used for the two ordinal dependent variables (*social_dist* and *mask*) due to their measurement with four ordered categories. Logistic regression was used for *App_use* as this variable only had two possible values (the App was either used or not used).

For regressions, *gender* was converted into two dummy variables: *female* (1 if female; 0 otherwise) and similarly *other gender*, measuring effects relative to males. Similarly, *region* was converted into two dummy variables *town* and *rural* (relative to city). *Vote* was converted into two dummy variables *voted* (1 if voted) and the control variable *votedP* (1 if prefer not to say), both relative to non-voters.

Separate regressions were performed for all three dependent variables in each of the three countries (9 regressions). All variables in Tables 1 and 2 were included as independent variables in all regressions, except *trust_info* which was only used to predict *App_use,* since provision of information does not apply for social distancing and masking.

Common method variance is reduced by several features. First, our dependent variables are all definitive actions rather than subjective attitudes. Second, we measure independent variables, such as the trust variables, with different descriptors as responses. Third, we include both *trust_govt* and *confidence_phs* in regressions so the effect of each variable is controlled for with the effect of the other.

## Results

Confidence in public health scientists predicts use of masks, social distancing and phone tracking applications use – albeit statistically insignificant for tracer phone application use in HK – providing broad support for Hypothesis H2 (Tables 4, 5 and 6). Confidence is higher than trust in government in USA and HK, but not Japan. Respondents using either masks or distancing are more likely to use both, and use of both is widespread in all countries.

Some findings are not as hypothesized. Trust in government *does* predict use of a phone tracking application, albeit statistically insignificant in Japan: as hypothesized (H1a). For predicting mask use and distancing however, trust in government is *negative* and insignificant in USA, and negative and significant in Japan and Hong Kong, falsifying parts of Hypothesis H1 (H1b and H1c). To the results in more detail.

A majority have confidence in public health scientists in the USA (69%) and HK (74%); a minority do so in Japan (40%) (Table 1). Trust in government is relatively low at 57% (USA), 45% (Japan), and 55% (HK). Confidence is significantly higher (*p* < 0.001) than trust in government in USA and HK, but the difference is not significant in Japan (*p* = 0.666). Moderate correlations exist between trust and confidence (*r* = 0.44 USA; 0.38 Japan; 0.29 HK). Confidence and trust in government are notably low in Japan relative to other liberal democracies, which finds other research support.

The use of a COVID-19 phone tracking application (*App_use*) is not common in any country, at 20% (USA), 24% (Japan), and 21% (HK; Table 3). Social distancing and mask use are common, practiced ‘often’ or ‘always’ by most respondents, ranging from 77% for social distancing in Japan, to 96% for mask use in HK. Relative to other countries, Japan has a higher app use and lower social distancing use. Mask use in Japan is lower than HK, but higher than the USA.

There are interesting differences in patterns of COVID-19 control mechanisms. *Social_dist* and *Mask* are highly correlated (*r* = 0.70) in the USA, but only moderately correlated in Japan (*r* = 0.51) and HK (*r* = 0.52). Hence, respondents using one of these are more likely to use the other. In contrast, USA respondents using the App have lower *social_dist* (mean = 3.00 using the App, compared to 3.35 for those not using the App, *p* < 0.001) and lower *mask* (3.13 compared to 3.50, *p* < 0.001). Results are similar in HK to the USA, for *social_dist* (3.33 and 3.64, *p* < 0.001) and *mask* (3.62 and 3.91, *p* < 0.001). In Japan, however, *social_dist* (3.16 and 3.00, *p* = 0.008) and *mask* (3.60 and 3.52, *p* = 0.179) are slightly *higher* for respondents using the App. These results suggest the use of a mask and social distancing are somewhat substitutional for COVID-19 App use in USA and HK, but complementary in Japan.

The likelihood of using the COVID-19 phone tracking App is positively associated with the three trust/confidence variables in all three countries, albeit with varying magnitude and statistical significance (Table 4). In the USA, *trust_govt* (B = 0.56, *p* < 0.001) and *confidence_phs* (B = 0.45, *p* = 0.001) significantly increase *App_use*, but *trust_info* has a relatively small and insignificant effect (B = 0.18, *p* = 0.135). In Japan, *confidence_phs* (B = 0.50, *p* < 0.001) has a strong relationship, but *trust_govt* (B = 0.16, *p* = 0.163) and *trust_info* (B = 0.13, *p* = 0.294) are insignificant. In HK, the opposite trend exists, with *trust_govt* (B = 0.93, *p* < 0.001) and *trust_info* (B = 0.89, *p* < 0.001) being significant, but *confidence_phs* (B = 0.089 *p* = 0.512) being insignificant. Removing *trust_info* from the regression sees *confidence_phs* significant for all countries. Hence, we find support for H1a in the USA and HK, but not in Japan; and find support for H2a in USA and Japan, but not HK. These effects are substantial. Increasing *trust_govt* in the USA by 1 point on the Likert scale, multiples the odds of using the COVID-19 App by approximately exp (0.56) = 1.8. Increasing *trust_govt* from 1 (not at all) to 4 (a great deal) has this effect three-fold, multiplying the odds of App use by 1.8^3^ = 5.4.

Government employees (*govt_empl*) are also significantly more likely to use the COVID-19 App in the USA, and to a lesser extent in Japan. In the USA, rural and older people are less likely to use the COVID-19 App. Conservative ideology significantly increases COVID-19 App use in HK, significantly decreases it in Japan, and has an insignificant effect in USA.

Confidence (*confidence_phs)* significantly increases social distancing and mask use in all countries, but the effect is smaller in Japan (Tables 5 and 6). Hence, we find support for *H2b* and *H2c* in all countries. *Trust_govt* lowers *social_dist* and *mask* in all countries, so we do not find support for H2b and H2c. Indeed, we find significant evidence that *trust_govt* lowers *social_dist* and *mask*, consistent with the negative correlations between these variables.

In contrast to *App_use*, government employees in all countries have significantly lower *social_dist* and *mask*. Both *social_dist* and *mask* are significantly higher for females (all countries), for voters (except *social_dist* in HK), and for older people (in USA and HK). Civic participation lowers *mask* in Japan and HK. *Social_dist* is significantly higher in USA rural areas but significantly lower in Japanese rural areas. Interestingly, conservative ideology is not significantly related to *social_dist* or *mask* in any country, but replacing this variable with party vote in the USA yields significant evidence that *social_dist* (B = 0.31, *p* < 0.001) and *mask* (B = 0.30, *p* < 0.001) are lower for Republicans than Democrats (but not *App_use*). This suggests political voting alignment rather than self-identified political ideology plays a part in the USA.

If trust and confidence are important for adoption of control measures, what predicts confidence and trust in government (Tables 7 and 8)? Confidence in public health scientists is significantly higher for respondents in all countries reporting higher civic participation and who had voted, and significantly lower for respondents who prefer not to disclose their gender. Furthermore, confidence in public health scientists is also significantly lower in Japan for respondents who prefer not to disclose whether they voted, and significantly lower in USA for respondents: who live in a rural area; are female; and more conservative. Confidence in public health scientists is also significantly higher in the USA for government employees.

Similar to *confidence_phs*, *trust_govt* is significantly higher when *partic_civic* and voting is higher, and in the USA is significantly higher for government employees, and significantly lower for females and rural residents. Here the similarities end. In the USA, *income* is positively – and *age* is negatively – associated with *trust_govt,* but *conservative* is not significant. In Japan, *govt_empl* and *female* are (respectively) positively and negatively associated with *trust_govt* (as in the USA), but *conservative* and *educ* are *positively* associated with *trust_govt*. In HK, the positive association between conservative ideology and *trust_govt* is stronger, and *income* is positively associated – and *educ* negatively – associated.

## Discussion and conclusion

A variety of methods of COVID-19 control are likely to be required from 2022 onwards. This is compounded by the variable availability and speed of vaccine roll-out, vaccine hesitancy, the rise of new COVID-19 variants, and the diminishing efficacy of vaccines over time. As such, the antecedents of phone tracking application and mask use, and social distancing, remain of vital research and policy concern, including for understanding future pandemic control. Drawing on online survey panels in three countries, with a broadly representative sample selected by quota, and using a variety of measures to predict use, we add to this understanding.

A central and policy-relevant contribution of our study is that higher levels of confidence in public health scientists significantly increases COVID-19 tracer application use, social distancing, and mask use, in the USA, Japan, and HK. The exception is COVID-19 Phone Application use in HK, where trust in the government, and trust in the security of digital information (a measure of privacy protection) provided to the government, are significant. Removing digital trust from the regression however, finds confidence significant for application use for all countries. Building and maintaining confidence in science – and public health scientists – provides a key tool to manage this and perhaps subsequent pandemics. Credible, effectively communicative – and independent – medical and scientific leaders may be central to pandemic control success [8, 26, 27]. Yet, we find in the United States, that confidence in public health scientists is lower in female, conservative, and rural dwelling respondents, providing support to those suggesting a cultural and ideological divide on COVID-19 control and perceptions of expertise in the United States, and supporting other studies suggesting Republican voting and conservative orientation are related to lower trust in science [7, 22]. Political and ideological factors then have important implications for public health and implementation of public health measures and outcomes, underpinning the importance of multi-disciplinary research and understanding in public health.

In recent studies, trust in government was a strong predictor of COVID-19 phone tracker app use [8], while other US studies suggested trust in government institutions was related to NPI use, and we expected this to be reflected in use of control methods in other countries [6]. We did find that phone tracking use is positively associated with trust in government, albeit statistically insignificantly so in Japan. But counter-intuitively and quite unexpectedly, we did *not* find a positive link between trust in government and use of masks and social distancing. Indeed, for Japan and HK, the results are significant and negative; in the USA the negative results are insignificant. Perhaps those who trust government are more likely to leave an abstract and beneficent government to save them from a pandemic, despite government policy suasion to act in certain ways; a plausible but not reassuring reading [28]. There may also be cultural or societal explanations. In Japan, high levels of personal hygiene are often claimed to be an aspect of Japanese culture [29, 30]. There is an established readiness to adopt preventative behavior such as mask wearing, supported by scientific leadership, and enforced through social conformity rather than government fiat; indeed, adoption of NPIs in Japan is voluntary rather than enforced by law as in some comparator countries [30, 31]. Trust in government may have little or no relation to this.

For the USA, this finding that trust in government is not significant could have a positive implication. Even in highly politically-contested times with variable trust in government and with members of a government perceived to be attacking scientific consensus and expertise and its proponents [32]; even then sufficient confidence in scientific expertise can stimulate compliance with pandemic control mechanisms. The trick will be to extricate perceptions of scientific expertise from partisan and ideological constraints; not an easy task by any means. One method of extrication might be through the use of an audience segmentation approach, including unpacking complex relationships of trust and race in relation to COVID-19 behaviors. This may be important in the US, where some that adhere to government recommendations lack trust in the US government [33], suggesting trust is only one factor in determining a person’s belief in the efficacy of, and compliance with, a given public health recommendation [34].

Our study generated some interesting findings, also of relevance for understanding public health policy. We find social distancing and mask use is practiced more widely than use of a COVID-19 App, especially in HK where individuals may be wary of providing information to a non-democratic government that may be perceived as not acting in their interests. Why this situation exists deserves further study, particularly with ubiquitous and relatively effortless smart phone and app use around the world. Perhaps the potential threats to privacy a tracking app carry may provide some part of the explanation, suggesting perceived security of information provided is important, and with this intuition supported by the link found between trust in government and security of digital information and phone application use. In collectivist Japan, the less easily-observable phone app use may lack the ‘virtue signalling’ of other forms of NPI such as mask wearing, and hence be less enforceable by societal norms and censure [30, 31]. Moreover, the development of the app in Japan by a commercial entity, was plagued by technical difficulties and bugs, which may have discouraged use. Trust, privacy protection, as well as technical efficacy, and perceptions of such, are likely to be important in citizen adoption of digital tools for public health in the future.

In the USA, Republican voters are less likely to practice social distancing and mask use, as are non-voters. This suggests successful efforts to depoliticise scientific expertise and medical interventions could have real effects in terms of success of public health measures. Women are higher users of masking and distancing. Despite a body of research suggesting government employees often exhibit pro-social motivations, while we find they are more likely to use the phone tracking applications, they are *less* likely to mask and practice social distancing. This is a counter-intuitive finding that deserves further investigation. While what predicts trust in government and confidence varies across countries, civic participation and voting are positively associated with both in all three countries, emphasising the interrelationship between socio-political factors, an engaged population, and effective policy and public health [4, 26].

This study has limitations, which suggest avenues for further research. We have not controlled for race, which a body of research suggests is important for trust in public health in the United States [7]. Differences between the US states might also be illuminating, given the considerable variation between them in approaches to COVID-19 management. In Hong Kong, with its highly divided political landscape, controlling for support for the CCP and first language might be of interest. Our trust in government measure is reasonably broad, and more finely targeted measures (such as disease control or health organizations) might have delivered different results [6]. However, a body of research finds different trust in government measures are often highly correlated [8]; moreover, focussing on particular health or policy organizations in the different countries would have limited the cross-country comparability and the generalizability of our study. Our use of an online survey method may exclude part of the population marginalized by a ‘digital divide’, particularly important in COVID-19 which can have unequal outcomes based on socio-economic status. But in a time of lockdowns, travel bans, and other controls, an online survey was likely the most pragmatic solution, particularly for a multi-country study. Our snap-shot study does not investigate changing relationships over time. While we are mainly interested in parsing policy-relevant empirical findings from our data, digestible in this single article, there are avenues for further theoretical and empirical investigation, that we have only touched on: in particular, the role of social capital and other antecedents of trust, confidence, and policy compliance [21]. Open government and transparency of government and health processes are likely to affect credibility of government communications and lead to better policy compliance, and deserve further investigation [14, 21, 30]. Many of our measures rely on self-reporting, which could overstate compliance. However, unless there are systematic biases in particular countries, this should not be such a problem for a cross-country study. Future studies using panel data, alternative methods of sample selection including non-digital ones, controlled experiments, expanded antecedents, qualitative interviews, and further comparative studies, might strengthen findings, including providing better understanding of causal relationships.

To conclude, in the face of continual anti-science rhetoric from elements of the population, COVID-19 conspiracy theories, and disinformation, this study reiterates the importance of establishing the credibility of – and confidence in – science, scientific expertise, and scientific experts, to encourage compliance with pandemic control methods. Trust in government and protection of privacy also matter in terms of encouraging the adoption of potentially highly invasive phone tracking applications, and perhaps other digital tools in public health. This confidence/trust has real world effects on the successful implementation of health policy measures. Building (and further investigating) both remains a worthy project for those working in the fields of policy and public health.

**Table 1:** Independent variables

	USA	Japan	HK
*trust_govt* (Government is generally trustworthy)			
1. strongly disagree	13%	16%	18%
2. disagree	29%	38%	27%
3. agree	45%	40%	48%
4. strongly agree	12%	5%	7%
*confidence_phs* (How much confidence do you have that public health scientists act in the best interests of the public?)			
1. not at all	10%	12%	7%
2. not too much	20%	48%	20%
3. fair amount	40%	35%	55%
4. a great deal	29%	5%	19%

**Table 2:** Control variables

	USA	Japan	HK
*partic_civic* (I am involved in local organizations, clubs, and volunteer activities)			
1. never	33%	56%	32%
2. seldom	28%	24%	32%
3. sometimes	27%	16%	31%
4 frequently	12%	4%	5%
*vote* (Did you vote in the last election?)			
Yes	65%	62%	64%
No	26%	30%	30%
prefer not to say	9%	9%	7%
*govt_empl* (I am a current or former government employee)^c^			
0 (no)	77%	87%	82%
1 (yes)	23%	13%	18%
*income* (What is your personal income before tax?)^b^			
1.^b^	15%	41%	11%
2. (US$15; JPY2000; HK$10)	11%	15%	4%
3. (US$25; JPY3000; HK$12)	10%	13%	8%
4. (US$35; JPY4000; HK$15)	12%	10%	14%
5. (US$50; JPY5000; HK$20)	16%	6%	17%
6. (US$75; JPY6000; HK$25)	13%	4%	12%
7. (US$100; JPY7000; HK$30	12%	3%	17%
8. (US$150; JPY8000; HK$40)	5%	2%	12%
9. ($US200; JPY9000; HK$60)	6%	6%	6%
*educ* (What is your highest education qualification?)			
1. High school incomplete or below	4%	2%	3%
2. High school completed	30%	30%	24%
3. Polytechnic or college certificate or diploma	13%	20%	12%
4. University or college undergraduate degree	32%	43%	49%
5. University postgraduate degree or above	20%	5%	11%
*age* (how old are you?)			
1. 18–24	13%	9%	13%
2. 25–34	17%	23%	24%
3. 35–44	18%	23%	27%
4. 45–54	18%	22%	22%
5. 55–64	16%	17%	12%
6. 65 +	18%	5%	2%
region (Do you live in a)			
City	51%	30%	81%
Town	31%	28%	13%
Rural	18%	42%	6%
*gender* (Identify your gender)			
Male	47%	48%	47%
Female	49%	51%	53%
prefer not to say^1^	4%	1%	0.3%
*conservative* (on a scale of 1 (more left or liberal) to 5 (more right or conservative), how would you describe your political beliefs?)			
1 Left Liberal	11%	3%	11%
2	17%	11%	19%
3	36%	62%	51%
4	16%	18%	16%
5 Right Conservative	20%	6%	4%
*trust_inf*o (How much trust do you have that information you provide to the government via website or email will be secure?)			
1. no trust	18%	29%	18%
2. some trust	38%	54%	40%
3. quite a bit of trust	30%	13%	34%
4. complete trust	15%	4%	8%

^a^Due to small numbers of responses, transgender (11 in USA and 3 in Japan) were combined with prefer not to say, giving a total prefer not to say of 43 (USA), 10 (Japan) and 3 (HK)

^b^Income ranges were specified in local currencies, with the lower bound indicated here (category 1 is less than the lower bound for category 2). Incomes are annual (USA and Japan) and monthly (HK). Values are in thousands

^c^Government employee was defined for all levels of government (e.g., in USA, federal, state, or municipal/local)

**Table 3:** Distribution of dependent variables

	USA	Japan	HK
*use_App* (Do you use any COVID-19 phone tracer applications?)			
1. no	80%	76%	79%
2. yes	20%	24%	21%
*social_dist* (Did you practice social distancing during the COVID-19 pandemic?)			
1. never	7%	8%	2%
2. seldom	15%	14%	8%
3. often	23%	44%	22%
4. always	56%	34%	69%
*mask* (Did you wear a face mask in public during the COVID-19 pandemic?)			
1. never	5%	3%	1%
2. seldom	11%	8%	2%
3. often	19%	21%	6%
4. always	65%	68%	90%

**Table 4:** Logistic regressions and Phone COVID-19 Tracking App (*App_use*)

Variable	USA		Japan		HK	
	B	P	B	P	B	p
*trust_govt*	0.56	0.000***	0.16	0.163	0.93	0.000***
*confidence_phs*	0.45	0.001***	0.50	0.000***	0.09	0.512
*partic_civic*	0.32	0.001**	0.06	0.519	0.35	0.001**
*voted*	0.33	0.169	0.14	0.451	0.37	0.082
*voteP*	-0.36	0.366	-0.14	0.675	-0.37	0.468
*govt_empl*	1.36	0.000***	0.44	0.043*	0.37	0.090
*Income*	0.03	0.549	0.12	0.002**	0.04	0.395
*Educ*	0.15	0.095	-0.06	0.481	0.07	0.466
*Age*	-0.37	0.000***	-0.10	0.111	-0.06	0.428
*Town*	-0.04	0.854	0.18	0.379	0.17	0.539
*Rural*	-0.72	0.022*	0.02	0.935	-0.06	0.885
*Female*	0.04	0.858	0.16	0.368	0.11	0.567
*gender prefer not to say*	0.53	0.303	0.33	0.702	-12.20	0.978
*Conservative*	-0.08	0.274	-0.25	0.011*	0.30	0.004**
*trust_info*	0.18	0.135	0.13	0.294	0.89	0.000***

^*^p < .05; **p < .01; ***p < .001

**Table 5:** Regressions predicting social distancing (*social_dist*)

Variable	USA		Japan		HK	
	B	P	B	P	B	p
*trust_govt*	-0.07	0.394	-0.21	0.012*	-0.34	0.000***
*confidence_phs*	0.51	0.000***	0.20	0.023*	0.26	0.006**
*partic_civic*	0.07	0.285	0.04	0.603	-0.15	0.066
*voted*	0.58	0.000***	0.67	0.000***	0.15	0.329
*voteP*	0.20	0.390	0.41	0.079	-0.29	0.303
*govt_empl*	-0.80	0.000***	-0.41	0.026*	-0.41	0.024*
*Income*	0.05	0.123	-0.01	0.803	0.04	0.259
*Educ*	-0.06	0.351	0.07	0.304	0.03	0.695
*Age*	0.34	0.000***	0.08	0.063	0.16	0.010*
*Town*	-0.03	0.858	-0.10	0.528	-0.18	0.374
*Rural*	0.44	0.018*	-0.29	0.045*	0.08	0.789
*Female*	0.52	0.000***	0.66	0.000***	0.41	0.004**
*gender prefer not to say*	-0.34	0.304	0.65	0.345	-1.34	0.263
*Conservative*	-0.08	0.158	0.15	0.053	-0.01	0.861

^*^p < .05; **p < .01; ***p < .001

**Table 6:** Regressions predicting mask use (*mask*)

Variable	USA		Japan		HK	
	B	P	B	P	B	p
*trust_govt*	-0.06	0.495	-0.18	0.052	-0.90	0.000***
*confidence_phs*	0.63	0.000***	0.21	0.041*	0.58	0.000***
*partic_civic*	0.03	0.706	-0.21	0.007**	-0.27	0.042*
*voted*	0.53	0.001***	0.74	0.000***	0.75	0.003**
*voteP*	0.73	0.005**	0.01	0.981	0.47	0.323
*govt_empl*	-0.75	0.000***	-0.50	0.011*	-1.29	0.000***
*Income*	0.02	0.483	0.00	0.881	-0.01	0.858
*Educ*	-0.01	0.934	0.09	0.233	-0.01	0.968
*Age*	0.30	0.000***	0.05	0.295	0.29	0.004**
*Town*	0.04	0.776	0.10	0.567	-0.08	0.825
*Rural*	0.25	0.201	0.17	0.307	-0.31	0.543
*Female*	0.72	0.000***	0.95	0.000***	0.49	0.034*
*gender prefer not to say*	-1.00	0.003**	0.04	0.955	11.85	0.000***
*Conservative*	-0.05	0.383	0.26	0.003**	0.06	0.666

^*^p < .05; **p < .01; ***p < .001

**Table 7:** Regressions predicting confidence in public health scientists (*confidence_phs*)

Variable	USA		Japan		HK	
	B	P	B	P	B	p
*partic_civic*	0.19	0.002**	0.19	0.007**	0.20	0.006**
*voted*	0.84	0.000***	0.34	0.016*	0.28	0.046*
*voteP*	0.01	0.950	-0.79	0.001**	-0.21	0.410
*govt_empl*	0.36	0.013*	0.03	0.869	0.01	0.964
*Income*	0.04	0.155	0.02	0.446	0.01	0.737
*Educ*	0.08	0.139	0.13	0.056	0.02	0.734
*Age*	0.06	0.115	0.08	0.097	0.00	0.943
*Town*	-0.24	0.073	0.05	0.773	-0.17	0.343
*Rural*	-0.51	0.002**	0.03	0.826	0.34	0.186
*Female*	-0.29	0.018*	0.09	0.512	-0.03	0.815
*gender prefer not to say*	-1.27	0.000***	-1.36	0.045*	-3.31	0.006**
*Conservative*	-0.29	0.000***	-0.03	0.663	0.11	0.101

^*^p < .05; **p < .01; ***p < .001

**Table 8:** Regressions predicting trust in government (*trust_govt*)

Variable	USA		Japan		HK	
	B	P	B	P	B	p
*partic_civic*	0.28	0.000***	0.23	0.001**	0.38	0.000***
*voted*	0.08	0.558	0.19	0.166	-0.27	0.052
*voteP*	0.03	0.902	-0.34	0.139	-0.23	0.378
*govt_empl*	0.64	0.000***	0.44	0.017*	0.30	0.075
*Income*	0.08	0.004**	0.05	0.077	0.08	0.015*
*Educ*	0.04	0.453	0.14	0.029*	-0.19	0.005**
*Age*	-0.16	0.000***	-0.02	0.692	0.05	0.347
*Town*	-0.21	0.115	0.17	0.280	0.17	0.331
*Rural*	-0.42	0.010*	-0.06	0.671	-0.51	0.054
*Female*	-0.36	0.003**	-0.27	0.044*	-0.02	0.857
*gender prefer not to say*	-0.57	0.076	-1.04	0.126	0.22	0.816
*Conservative*	-0.01	0.822	0.24	0.002**	0.61	0.000***

^*^p < .05; **p < .01; ***p < .001

## Data Availability

The datasets used and/or analysed during the current study available from the corresponding author on reasonable request.
